# Smartphone-Based Localization for Passengers Commuting in Traffic Hubs

**DOI:** 10.3390/s22197199

**Published:** 2022-09-22

**Authors:** Francisco Jurado Romero, Estefania Munoz Diaz, Dina Bousdar Ahmed

**Affiliations:** German Aerospace Center (DLR), Institute of Communications and Navigation, Oberpfaffenhofen, 82234 Wessling, Germany

**Keywords:** indoor navigation, pedestrian dead-reckoning, urban navigation, indoor landmark detection

## Abstract

Passengers commute between different modes of transportation in traffic hubs, and passenger localization is a key component for the effective functioning of these spaces. The smartphone-based localization system presented in this work is based on the 3D step and heading approach, which is adapted depending on the position of the smartphone, i.e., held in the hand or in the front pocket of the trousers. We use the accelerometer, gyroscope and barometer embedded in the smartphone to detect the steps and the direction of movement of the passenger. To correct the accumulated error, we detect landmarks, particularly staircases and elevators. To test our localization algorithm, we have recorded real-world mobility data in a test station in Munich city center where we have ground truth points. We achieve a 3D position accuracy of 12 m for a smartphone held in the hand and 10 m when the phone is placed in the front pocket of the trousers.

## 1. Introduction

Traffic hubs are dedicated public spaces, such as train stations or airports, where passengers commute from one means of transport to another. The localization of passengers in traffic hubs is a key aspect to the effective functioning of these spaces.

In most of the cases, traffic hubs are roofed or underground and the use of Global Navigation Satellite Systems (GNSS) is not possible. Nontheless, passenger localization can be performed by means of infrastructure-dependent technologies, such as WiFi [1,2], Bluetooth [3] or Ultra-Wideband (UWB) [4]. In these cases, a number of access points need to be deployed along the traffic hub.

Furthermore, infrastructure-free technologies are also a suitable solution for indoor positioning. Pedestrian dead-reckoning performed with inertial sensors is of high interest because of the possibility of providing localization without violating privacy, unlike camera-based systems [5,6]. Suitable locations of the inertial sensors are the torso [7], the shoe [8,9] or the pocket [10,11], while the inertial sensors embedded in the passenger’s smartphone can be also used.

Nevertheless, smartphone-based localization of passengers in traffic hubs is challenging because the smartphone is not attached to the body and different carrying modes are possible, such as handheld, in the pocket or in a backpack [12]. Moreover, the sensors embedded in smartphones usually have low quality.

Information from maps can be used to reduce position error [13,14]. The main inconvenient of this method is that accurate maps of traffic hubs are usually not publicly available. Another approach to reduce the error is using landmarks. These are recognizable and observable characteristic elements present in the environment. An example of landmarks used to correct position error are corridors, corners and stairs [15,16,17]. The authors in [18] detect escalators and lifts, which can be used as well as landmarks.

The main contribution of this work is an infrastructure-free indoor localization system based on pedestrian dead-reckoning using the sensors embedded in a smartphone. In addition, we propose the use of staircases and lifts in the traffic hub as landmarks to reduce position error and the use of ground truth points measured in a real station to evaluate the localization system.

This paper is organized as follows: The data collection campaign performed to evaluate the system is introduced in Section 2. Section 3 presents the proposed smartphone-based passenger localization system. The evaluation of the system is presented in Section 4. Finally, conclusions are drawn in Section 5.

## 2. Data Collection

**Figure 1:**
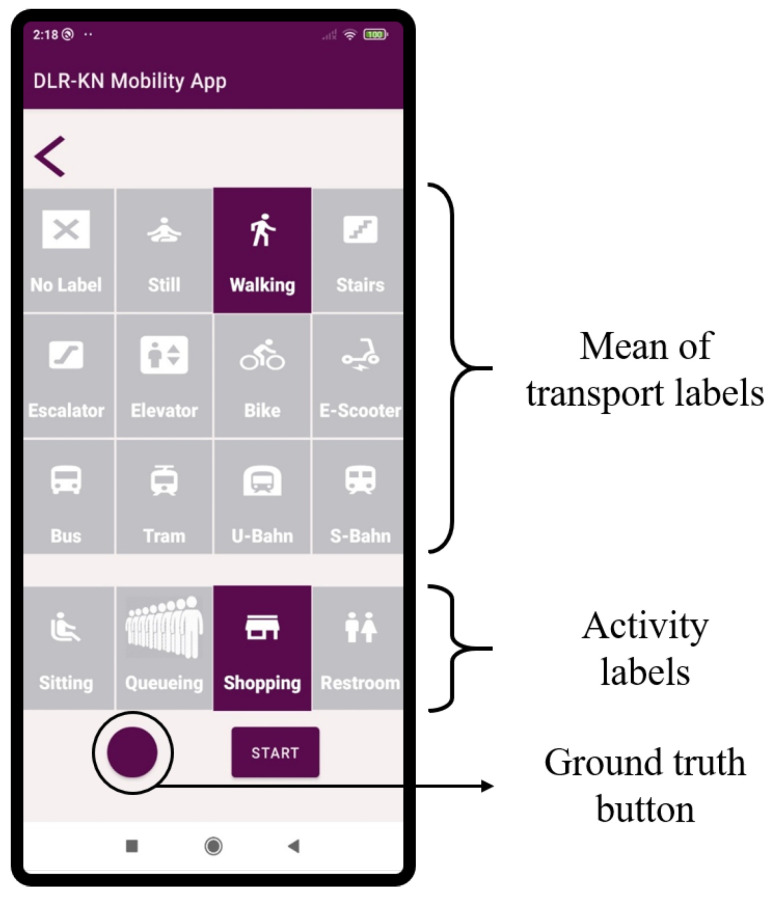
Screenshot of the smartphone app used to record the mobility data.

We have developed a smartphone app to record and label mobility data. We record the data from the accelerometer, gyroscope, magnetometer and barometer of the smartphone at a sampling frequency of 100 Hz. Figure 1 shows an screenshot of the smartphone app that has been used to gather the mobility data.

With the smartphone app, we indicate with different labels the means of transport the passenger is using at each moment, i.e., if the passenger is walking, riding a bike or an e-scooter or using a bus, tram, subway or train. To further analyze the use of infrastructure in a traffic hub, we also indicate the use of staircases, escalators, elevators, benches, shops and restrooms, as well as activities related to the use of infrastructure, such as waiting in a queue. Our app also includes a button to mark Ground Truth Points (GTP). These points are used to compute the accuracy of the localization system presented in this work.

**Figure 2:**
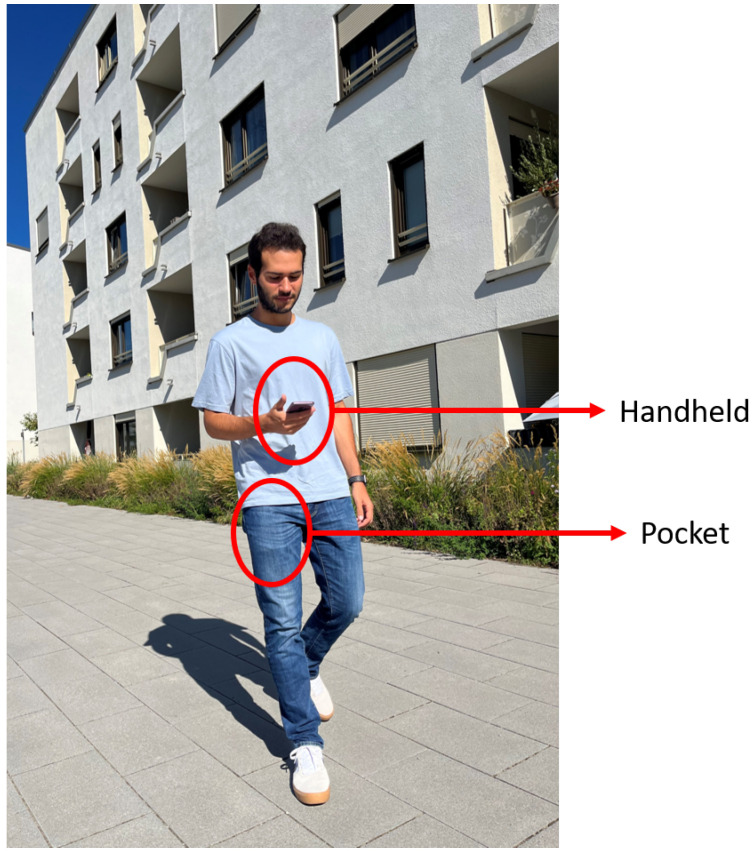
Experimental set-up. The volunteers carry two smartphones in different positions: in the front pocket of the trousers and held in the hand.

The data collection campaign has taken place during four months and six different volunteers have been involved taking measurements twice a week. During the experiments, the volunteers carried a total of two smartphones, which were located in the trousers front pocket and held in the hand. The smartphones used were a Google Pixel 4 and a Samsung Galaxy S20, respectively. The volunteers have labeled the mobility data with the smartphone held in the hand. Both smartphones are synchronized to record data and the labels are transfered to the pocket smartphone. The set-up of the smartphones is shown in Figure 2.

A total of four different trips have been designed so the volunteers arrive at our test station with one means of transport, make use of the infrastructure of the test station and leave the station with a different means of transport.

**Figure 3:**
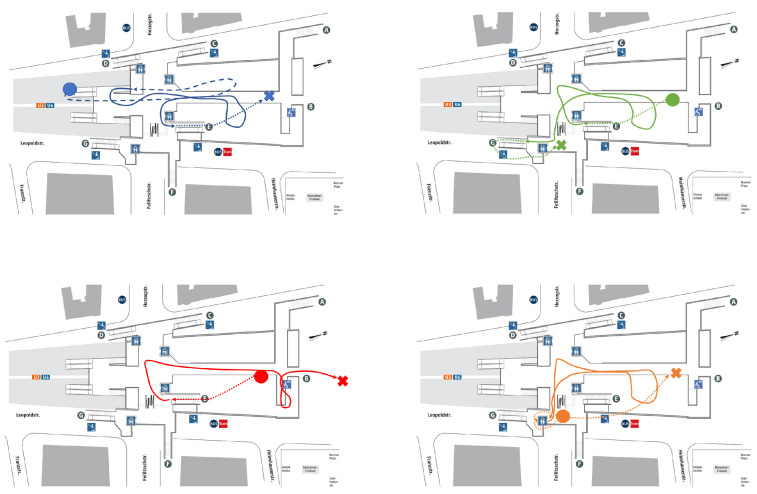
Designed 3D trajectories in the test station. The dots indicate where the trajectories start and the crosses where they end. The dashed lines indicate the lower floor; the solid lines indicate the main floor; the dotted lines indicate the upper floor.

In this work, we only make use of the parts of the trips that take place inside the test station, where we have recorded a total of 30 h of data. Figure 3 depicts the walks that the volunteers have recorded inside the test underground station.

**Figure 4:**
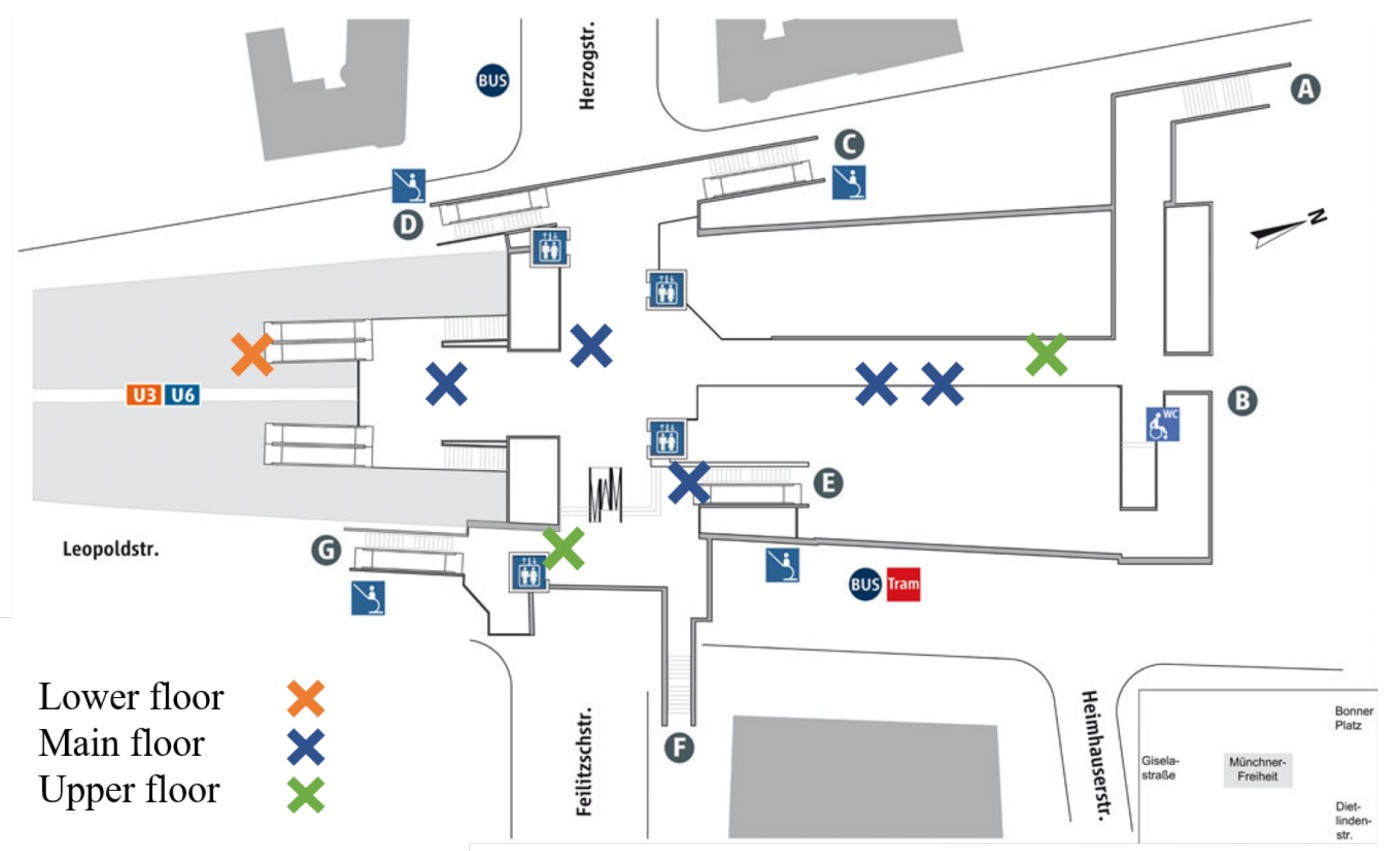
Map of the Ground Truth Points (GTP) in the test station.

Thanks to our collaboration with the local mobility provider of the city of Munich, we have measured with centimeter accuracy eight different reference points in our test station. Each of these points is used as GTP. The GTPs are distributed in the station among all floors: one in the platform floor, five in the main floor and two in the upper floor, as shown in Figure 4. The position of each GTP has been measured with a Leica tachymeter, model TPS 1200 [19]. During the data collection, the GTPs are visited and marked over 3000 times.

**Figure 5:**
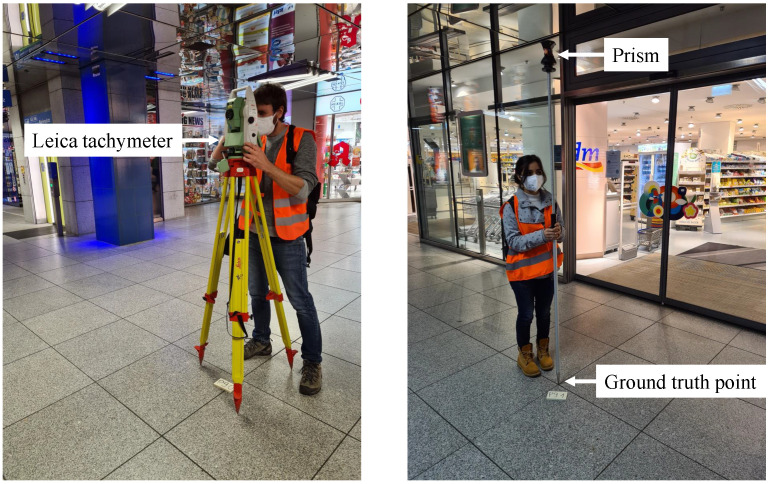
(**Left**) The Leica tachymeter station which measures the distance to the prism. (**Right**) The prism placed at a pre-defined GTP.

Figure 5 shows an example of the measurement of a GTP position inside the station. The Leica tachymeter measures the distance to the prism, which is positioned at the GTP and estimates its coordinates. As Figure 5 shows, the GTP and the prism are not at the same height. Nonetheless, the height difference is compensated by the Leica tachymeter when estimating the position of the GTP.

We assume that the volunteer’s heading is the same as the smartphone’s heading. We align the GTP coordinate system and the passenger trajectory coordinate system by placing the origin of the walk in a known GTP used as starting point of the walk. In addition, the walks start with a known heading of the passenger.

We define the error, *e*, as the Euclidean distance between the estimated position ($p_{e}=\left(x_{e},y_{e},z_{e}\right)$) and reference position ($p_{r}=\left(x_{r},y_{r},z_{r}\right)$) of the GTP.

(1) $$e=\sqrt{{\left(x_{r}-x_{e}\right)}^{2}+{\left(y_{r}-y_{e}\right)}^{2}+{\left(z_{r}-z_{e}\right)}^{2}}$$

In addition, we have defined a total of nine landmarks in the station, which are all the stairs and lifts used during the walks. These landmarks are visited a total of 338 times and are used to correct the error of the localization system.

## 3. Passenger Localization

The smartphone-based localization system presented in this work is based on the 3D step and heading approach. This system is valid for two different carrying modes, i.e., the position in which the passenger carries the smartphone:

- In the front pocket of the trousers;
- Held in the hand.

**Figure 6:**
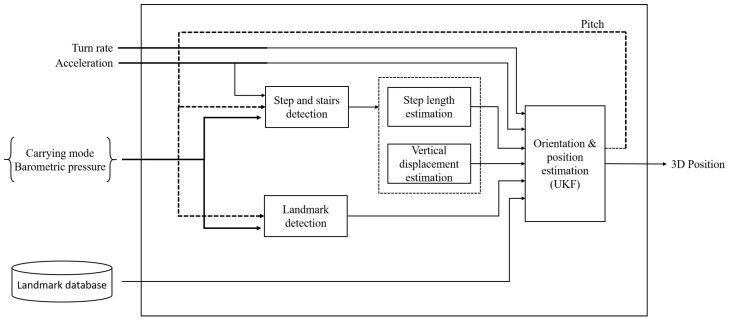
Block diagram of the smartphone-based localization system with several possible carrying modes.

In this work, we evaluate separately the pocket and handheld carrying modes, since we do not consider trajectories in which the carrying mode is changed. A block diagram of the localization system is presented in Figure 6.

### 3.1. Step Detection

**Figure 7:**
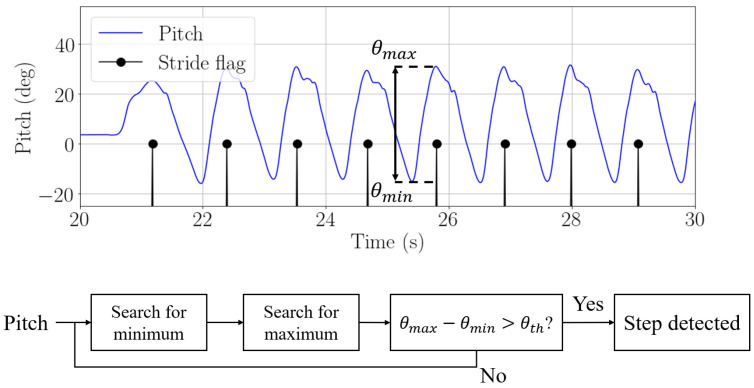
Pitch estimation (blue) and detected strides (black) when the passenger carries the smartphone in the front pocket of the trousers and algorithm for step detection.

The step detection algorithm is adapted depending on the carrying mode. For the pocket, we use the step detection algorithm presented in [20]. This step detection algorithm was first demonstrated with external inertial sensors and it can also be used with the inertial sensors embedded in a smartphone. When the smartphone is introduced in the front pocket of the trousers, the pitch angle mirrors the movement of the leg. A new stride is detected every time a maximum in the pitch angle occurs. Since the smartphone is in the pocket in one leg, the pitch angle allows to identify strides, i.e., one every two steps. Figure 7 depicts the described step detection based on the pitch angle and the algorithm to detect steps.

**Figure 8:**
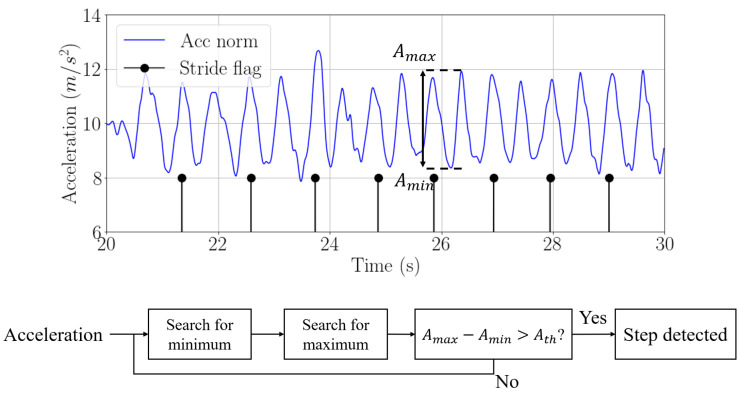
Norm of acceleration (blue) and detected strides (black) when the passenger carries the smartphone in the hand and algorithm for step detection.

When the smartphone is held in the hand, we implement a step detector based on the movement of the hand while walking. In this case, the passenger’s step is detected every time a peak in the acceleration occurs [21]. The acceleration is low-pass filtered with a 5-s moving average window to obtain the peaks of acceleration due to the steps of the passenger. In order to make both step detection algorithms analogous, we also detect one every two steps when the smartphone is held in the hand. Figure 8 shows the step detection based on the norm of acceleration and the algorithm to detect steps. Both walks in Figure 7 and Figure 8 have been recorded simultaneously by the same passenger carrying two phones, in the pocket and held in the hand, respectively.

### 3.2. Step Length Estimation

When the smartphone is placed in the pocket, we estimate the step length based on the amplitude of the pitch angle. This step length estimation is based on the linear relationship between the amplitude of the pitch and the step length [10]:

(2) $$s_{p}=a_{p}\cdot \delta_{\theta }+b_{p},$$

where $s_{p}$ represents the step length estimation when the phone is in the pocket, $\delta_{\theta }$ represents the amplitude of the estimated pitch angle and $a_{p}$ and $b_{p}$ are two parameters that can be adjusted for every user, as described in [10].

When the smartphone is held in the hand, we implement a step length estimator based on the amplitude of the norm of acceleration. In this case, the step length can be estimated based on a linear relationship between the amplitude of the acceleration peaks and the step length of the passenger:

(3) $$s_{h}=a_{h}\cdot \delta_{A}+b_{h},$$

where $s_{h}$ represents the step length estimation when the phone is held in the hand, $\delta_{A}$ represents the amplitude of the recorded acceleration and $a_{h}$ and $b_{h}$ are two parameters that can be adjusted for every user in the same way as the pocket step length estimation.

### 3.3. Vertical Displacement Estimation

Passengers change the floor by using staircases and elevators. In order to perform 3D localization, we implement different methods to estimate the vertical displacement when the passenger carries the smartphone in the front pocket of the trousers and held in the hand.

**Figure 9:**
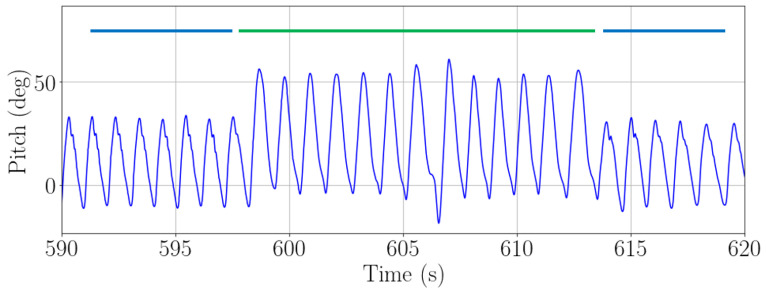
Detection of vertical displacement in stairs with the pitch angle. The green solid line above the pitch estimation indicates the moment when the passenger is climbing a staircase. The blue solid line indicates the moment when the passenger is walking on a horizontal surface.

When a passenger carries the smartphone in the front pocket of the trousers, the amplitude of the pitch angle can be used to estimate the vertical displacement of the passenger. This method was presented in [10] and is only to be used when the smartphone is placed in the pocket, since it reflects the movement of the leg. This method allows to detect all staircases that the passenger walks and also allows differentiating if the passenger is going upstairs or downstairs. Figure 9 shows an example of the pitch estimation when the smartphone is placed in the front pocket of the trousers and the passenger walks a staircase.

When a staircase is detected with the smartphone in the pocket, the vertical displacement is set to a deterministic value, which corresponds with the height of two steps, since we detect one every two steps.

Moreover, the barometric pressure can also be used to detect changes in height. This method can be used when the passenger is carrying the smartphone in the trousers and when the smartphone is held in the hand.

However, the barometer also records changes in the barometric pressure due to intense air flows, such as the air flows generated by the trains arriving or leaving the station or near the exits. The variations in barometric pressure due to air flows in the traffic hub have higher frequency components than the variations due to the vertical displacement of the passenger. Therefore, to be able to extract relevant information about the passenger’s vertical displacement, the barometric pressure must be low-pass filtered to discard the variations due to air flows. We filter the barometric pressure with a 2-s moving average window.

**Figure 10:**
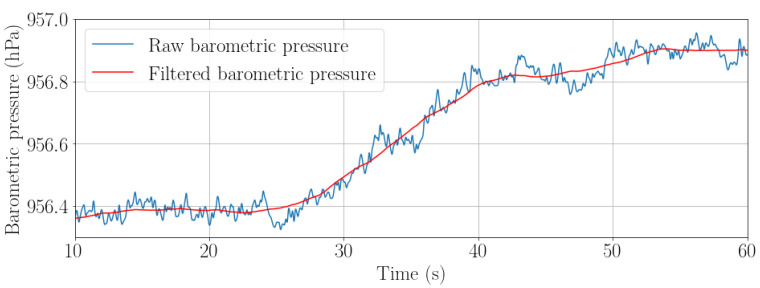
Raw (blue) and filtered (red) barometric pressure recorded while a passenger is climbing a staircase.

Figure 10 depicts how the raw signal recorded with the barometer changes rapidly due to the air flows in the station and the result after filtering the signal to obtain the information of the vertical displacement.

Because of the barometric pressure being low-pass filtered, changes in height smaller than 1.5 m are not detected and, therefore, staircases that are shorter than 1.5 m high are not detectable in this environment using the barometer.

**Figure 11:**
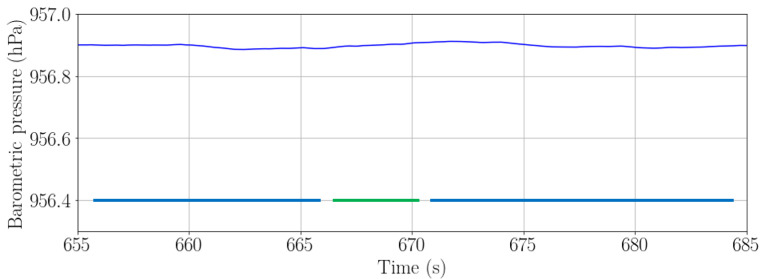
Barometric pressure when a passenger is walking a 1.2 m high staircase with a handheld smartphone. The green solid line indicates the moment when the passenger is climbing a staircase. The blue solid line indicates the moment when the passenger is walking on a horizontal surface.

**Figure 12:**
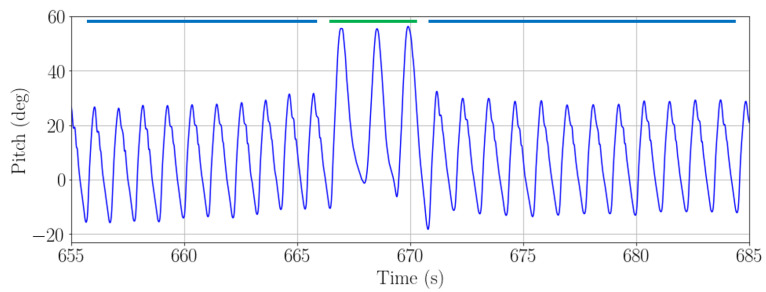
Pitch amplitude when a passenger is walking a 1.2 m high staircase with a smartphone in the front pocket of the trousers. The green solid line indicates the moment when the passenger is climbing a staircase. The blue solid line indicates the moment when the passenger is walking on a horizontal surface.

Figure 11 shows the barometric pressure recorded with a handheld smartphone and Figure 12 the pitch angle recorded with a smartphone in the front pocket of the trousers. In both figures, the passenger is carrying simultaneously both smartphones while walking a staircase smaller than 1.5 m high. In the figures, the green line represents the time when the passenger is walking the staircase. Both walks have been recorded simultaneously by the same passenger carrying both smartphones, held in the hand and in the pocket, respectively.

**Figure 13:**
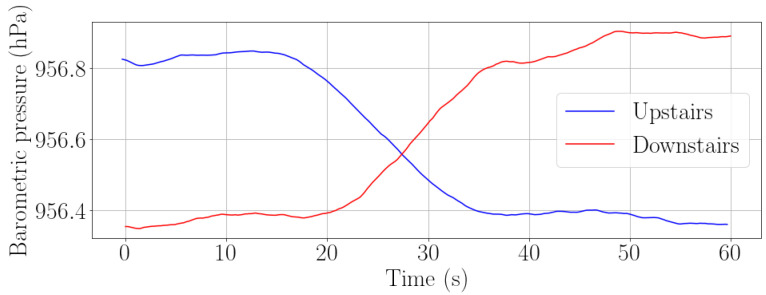
Barometric pressure when a passenger is going upstairs (blue) and downstairs (red).

The barometric pressure will be greater when the passenger is in a lower floor and lower when the passenger is in an upper floor. Therefore, this method also allows to determine if the passenger is going upstairs or downstairs. To determine the direction in which the passenger is moving, we observe the sign of the slope of the barometric pressure signal. When the slope is positive, the passenger is going downstairs, whereas the slope being negative means that the passenger is going upstairs. In Figure 13, both the barometric pressure signal when a passenger moves up and downstairs is shown.

**Figure 14:**
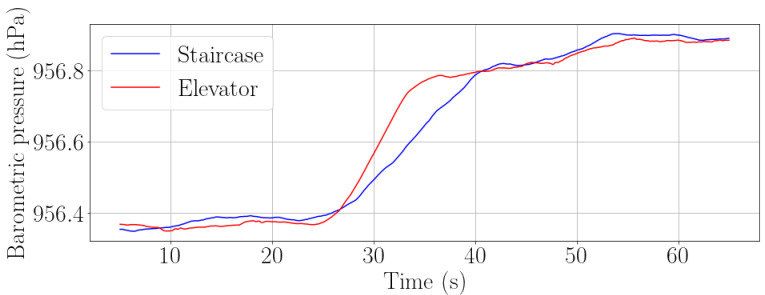
Comparison of the recorded barometric pressure when a passenger is using a staircase (blue) and an elevator (red) at walking speed (around 3.5 km/h).

The barometer allows to detect both staircases and elevators. In order to distinguish if a passenger is using a staircase or an elevator, we observe the changing rate over time of the barometric pressure. Assuming that the passenger is moving at walking speed, namely, around 3.5 km/h, the barometric pressure will change faster over time while the passenger is using an elevator than when using the staircase. Figure 14 shows how the barometric pressure changes more rapidly when using the elevator than when walking the stairs.

When an elevator is detected, the vertical displacement is set to a fixed value according to the average speed of the elevators in the traffic hub. On the contrary, if a stair is detected, the vertical displacement will be set to another deterministic value, which corresponds to the physical height of the steps, every time a step is detected.

### 3.4. Landmark Detection and Association

Landmarks are characteristic elements that remain always in the same position and can always be identified in the same way. In a traffic hub, staircases and elevators are elements that always stay in the same position and can be identified with the aforementioned methods to detect the passenger’s vertical displacement.

**Figure 15:**
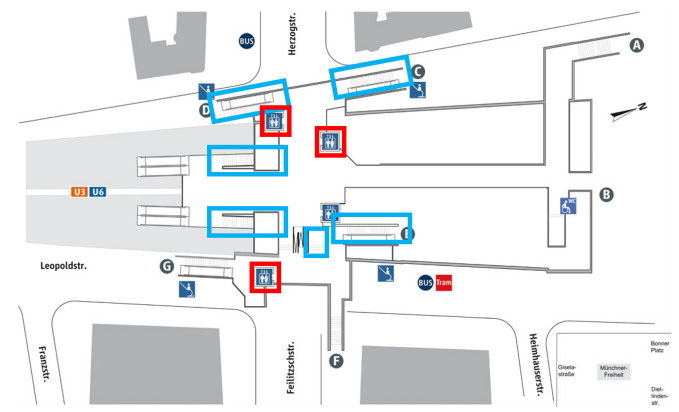
Landmark position in the Münchner Freiheit underground station. The blue rectangles indicate the position of staircases. The red rectangles indicate the position of elevators.

We have created a map which contains the exact location of every staircase and elevator in the test station. The information of this map is stored in a landmark database. Figure 15 shows the location of the staircases (marked in blue) and elevators (marked in red) in the Münchner Freiheit underground station.

**Figure 16:**
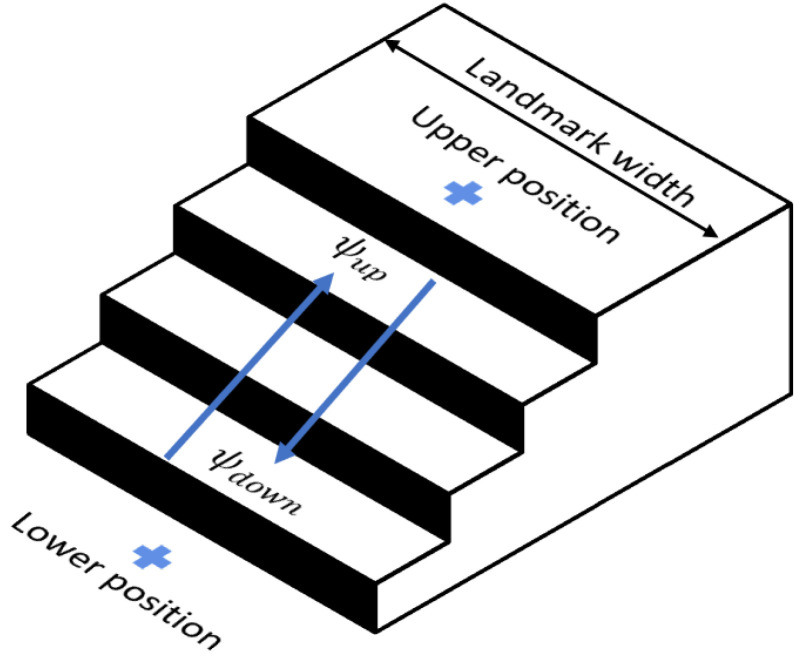
Example of the upper and lower parts of a landmark in an underground station.

Since staircases and elevators are placed in between two different floors, they are represented by two positions in the database, one corresponding to the upper floor and another corresponding to the lower floor. In the case of staircases, their orientation in the map defines the heading, ψ, in which a passenger walks the stairs. Elevators move vertically and, therefore, their orientation cannot define the passenger’s heading. Figure 16 depicts the landmarks’ physical parameters that are stored in the database and Table 1 shows an example of the information related to each landmark.

When the landmarks are detected with the algorithms presented in Section 3.3, i.e., detecting that the passenger is using a staircase or an elevator, they are compared with the landmarks stored in the database and associated with the closest one. Once associated, the landmarks can be used to correct the estimated passenger position and heading.

First, when the landmark is detected, the vertical position of the passenger is compared with the vertical position of the landmark. By doing this, we only target to associate the landmarks that are in the same floor as the passenger when the landmark is detected.

After selecting the landmarks that are placed in the same floor as the passenger, the Euclidean distance between the passenger’s position and the selected landmarks is computed. The associated landmark will be the the closest to the passenger’s position when the landmark is detected.

**Figure 17:**
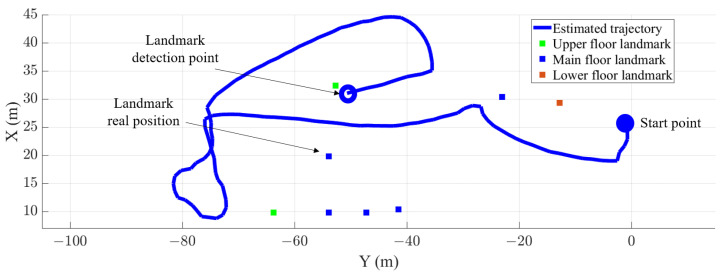
Estimation of a passenger trajectory in a traffic hub while walking through the main floor of the test station (solid blue) and the position of the landmarks (squares). The green squares represent the landmarks in the upper floor, the blue squares represent the landmarks in the main floor and the orange squares represent the landmarks in the lower floor of the test station.

Figure 17 shows the trajectory of a passenger in the main floor of the test station in solid blue. The landmarks’ position is marked with squares: green for the landmarks in the upper floor, blue for the landmarks in the main floor and orange for the landmarks in the lower floor. The point of the trajectory when a landmark is detected in the trajectory is marked with a circle as the landmark detection point.

### 3.5. Orientation and Position Estimation

**Figure 18:**
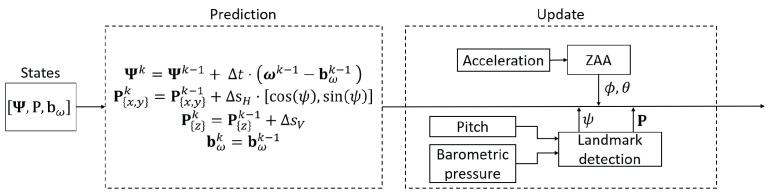
Architecture of the UKF used for orientation estimation.

We estimate the passenger’s orientation and position with the same UKF regardless of the carrying mode. The architecture of the UKF is shown in Figure 18.

The state vector, x, is composed of three elements:

- The Euler angles Ψ=[ϕ,θ,ψ];
- The position vector $P=[p_{x},p_{y},p_{z}]$;
- The gyroscope bias $b=[b_{x},b_{y},b_{z}]$.

(4) $$x=[\phi ,\theta ,\psi ,p_{x},p_{y},p_{z},b_{x},b_{y},b_{z}]$$

For the initialization of the state vector, the volunteers stand still for 5 s, where the initial roll and pitch are computed using the Zero Acceleration Assumption (ZAA), described in Equation (11). The initial gyroscope biases are extracted from the datasheet [22]. The initial position and heading are initialized for each walk taking into account the GTP where the walk starts.

In the prediction phase, the Euler angles prediction is based on the integration of the measured rotation of the smartphone, ω:

(5) $$\Psi^{k}=\Psi^{k-1}+\Delta t\cdot \left(\omega^{k-1}-b^{k-1}\right),$$

where $\omega =[\omega_{x},\omega_{y},\omega_{z}]$ represents the turn rate and Δt represents the sampling period.

The passenger’s position prediction is based on the step and heading algorithm, which follows:

(6) $$\begin{array}{c} p_{x}^{k}=p_{x}^{k-1}+\Delta s_{h}^{k-1}\cdot \cos\left(\psi^{k-1}\right) \end{array}$$

(7) $$\begin{array}{c} p_{y}^{k}=p_{y}^{k-1}+\Delta s_{h}^{k-1}\cdot \sin\left(\psi^{k-1}\right) \end{array}$$

(8) $$\begin{array}{c} p_{z}^{k}=p_{z}^{k-1}+\Delta s_{v}, \end{array}$$

where $p_{x}$ and $p_{y}$ represent the passenger’s position in X and Y, respectively, and $\Delta s_{h}$ represents the estimated step length of the passenger. The 3D positioning is solved with the information of the vertical displacement, $\Delta s_{v}$.

The gyroscope bias is modeled as constant between two consecutive instants since the sampling frequency of the system is high (100 Hz):

(9) $$b^{k}=b^{k-1}$$

The direction of movement of the passenger is considered in this work to be the heading of the smartphone.

**Figure 19:**
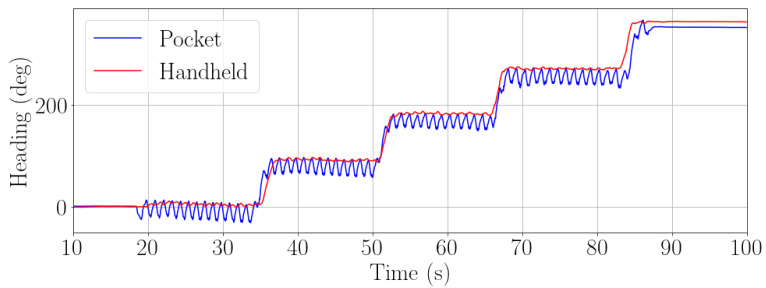
Estimation of heading in a square-like trajectory for two smartphone carrying modes: pocket (blue) and handheld (red). The walk has been recorded by using simultaneously two smartphones.

Figure 19 shows the estimated heading for pocket and handheld carrying modes over a square-like trajectory. In this case, the same passenger is carrying both smartphones simultaneously. The ripple in the heading estimation of the pocket carrying mode corresponds to the movement of the hip. This movement is not noticeable in the handheld carrying mode.

**Figure 20:**
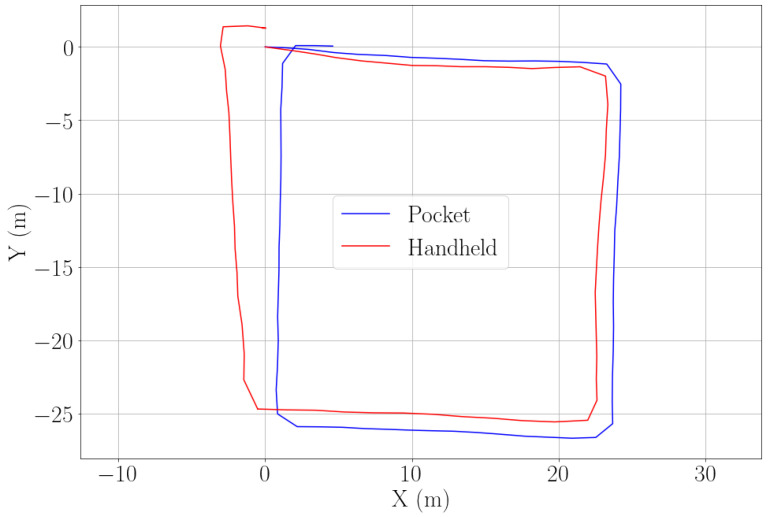
Estimation of position in a square-like trajectory for two smartphone carrying modes: pocket (blue) and handheld (red). The walk has been recorded by using simultaneously two smartphones.

Figure 20 shows the estimated position for pocket and handheld carrying modes over the same square-like trajectory. Again, the passenger is carrying both smartphones simultaneously.

In the update stage, we implement three different updates in the UKF:

- Zero Acceleration Assumption update (ZAA): This update is based on the detection of periods when the acceleration is zero or quasi-zero. During these periods of time, the accelerometers only measure gravity and the roll and pitch angles can be estimated as:
  (10) $$\begin{array}{c} \phi_{z}^{k}=\arctan\left(\frac{\alpha_{y}^{k}}{\alpha_{z}^{k}}\right) \end{array}$$
  (11) $$\begin{array}{c} \theta_{z}^{k}=\arctan\left(\frac{-\alpha_{x}^{k}}{\sqrt{{\alpha_{y}^{k}}^{2}+{\alpha_{z}^{k}}^{2}}}\right) \end{array}$$
  where $\phi_{z}^{k}$ and $\theta_{z}^{k}$ are the roll and pitch angles, respectively, with which to update the UKF and $\alpha_{x,y,z}^{k}$ is the acceleration measured in the smartphone in X, Y and Z directions at instant *k*.
- Landmark based passenger’s position update: This update is based on the detection of landmarks in a traffic hub described in Section 3.4. Once a landmark is detected, its position is associated to the passenger’s position. The position of the landmark is taken from the landmark database and it is used to update the passenger’s position as follows:
  (12) $$P_{l}^{k}=P_{R_{l}}^{DB},$$
  where $P_{l}^{k}$ represents the position that will be used to update the UKF and $P_{R_{l}}^{DB}$ is the reference position of the associated landmark that is taken from the landmark database.
  The positon is updated only once when the passenger walks out of the landmark, i.e., when the passenger reaches the upper or lower part of a staircase or when walking out of an elevator, since the landmark database describes the landmark only with their upper and lower positions.
  The position update is performed towards the position in the database. However, there is an uncertainty in this update related to the physical dimensions of the landmark. In this case, we consider the landmark width as the uncertainty for updating the passenger’s position in X and Y coordinates, and the step height as the uncertainty for updating the passenger’s position in Z coordinate.
- Landmark based passenger’s heading update: This update is also based on the detection of landmarks described in Section 3.4. When a passenger walks a staircase, the passenger’s heading is bounded by the direction in which the staircase is oriented. Therefore, the physical orientation of the staircase can be used to update the passenger’s heading as follows:
  (13) $$\psi_{l}^{k}=\psi_{R_{l}}^{DB},$$
  where $\psi_{l}^{k}$ represents the heading that will be used to update the UKF and $\psi_{R_{l}}^{DB}$ is the reference heading of the associated landmark.
  The heading update is performed constantly while climbing the stairs. This update also has an uncertainty related to the physical width of the staircase, since the passenger can walk the staircase diagonally from one side to the other. We consider the uncertainty of the heading update to be the difference between the heading of a passenger walking the stairs diagonally and the heading when walking the stairs in a straight line.

**Figure 21:**
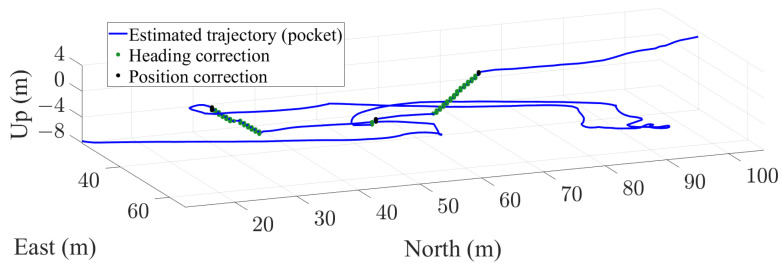
Estimation of a trajectory in a traffic hub with the smartphone in the front pocket of the trousers. The green and black dots represent, respectively, the points where heading and position updates are applied.

**Figure 22:**
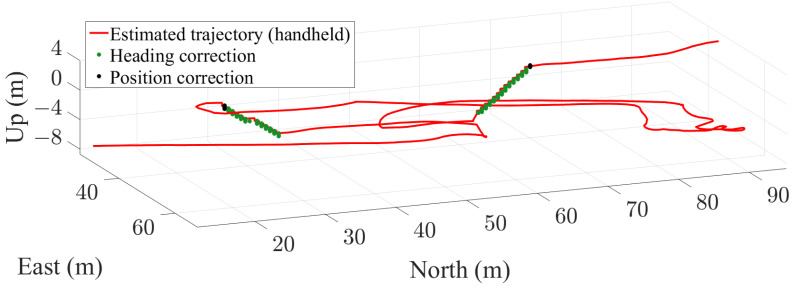
Estimation of a passenger’s trajectory in a traffic hub with a handheld smartphone. The green and black dots represent, respectively, the points where heading and position updates are applied.

Figure 21 and Figure 22 show the estimation of a passenger’s trajectory in a traffic hub with the described UKF for pocket and handheld carrying modes, respectively. Both trajectories have been recorded simultaneously.

The trajectory is walked over three different floors of the test underground station using three different staircases. In the trajectory, a staircase smaller than 1.5 m is climbed at coordinates [East,North,Up]=[48.2,49.5,−3.1] m. This staircase is detected and used for correcting when the smartphone is placed in the pocket. However, the staircase smaller than 1.5 m is not detected with the handheld smartphone. Nevertheless, for the handheld smartphone, the accumulated error in height is corrected when the next staircase is detected.

## 4. Evaluation

In this section, we will evaluate the accuracy of the localization system, which will be done in two main parts. First, the localization algorithms based on the pocket smartphone and handheld smartphone will be evaluated without the landmark-based error correction. Secondly, the same evaluation will be made but including the landmark-based error correction.

For the evaluation, we use 30 h of walking labeled data in the database presented in Section 2. The average length of the walks is 20 min and we use data from six different volunteers. Throughout the walks, the different GTPs used for computing the error are visited over 3000 times. The average time between two consecutive GTPs is 30 s.

The error is computed at each of the visited GTP in the walks, and it is defined as the Euclidean distance between the estimated position and the real position of the GTP, as introduced in Equation (1) in Section 2.

**Figure 23:**
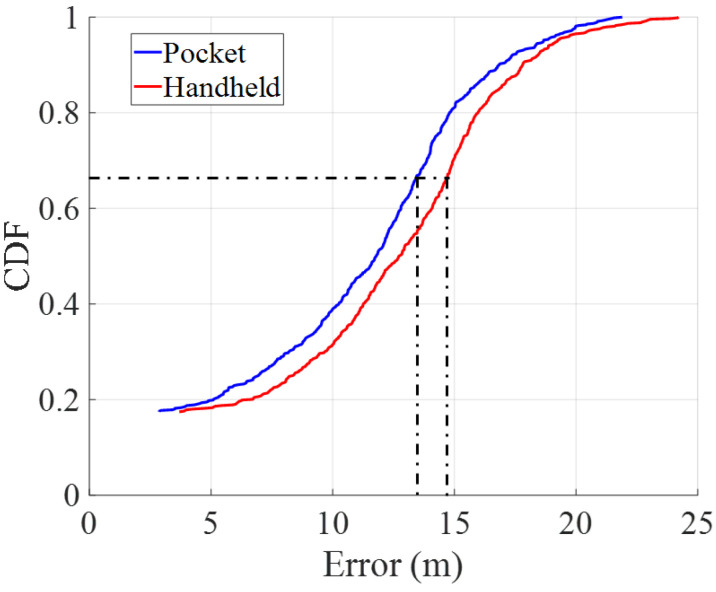
Cumulative density functions of the accumulated error for pocket (blue) and handheld (red) carrying modes without landmark-based position correction.

**Figure 24:**
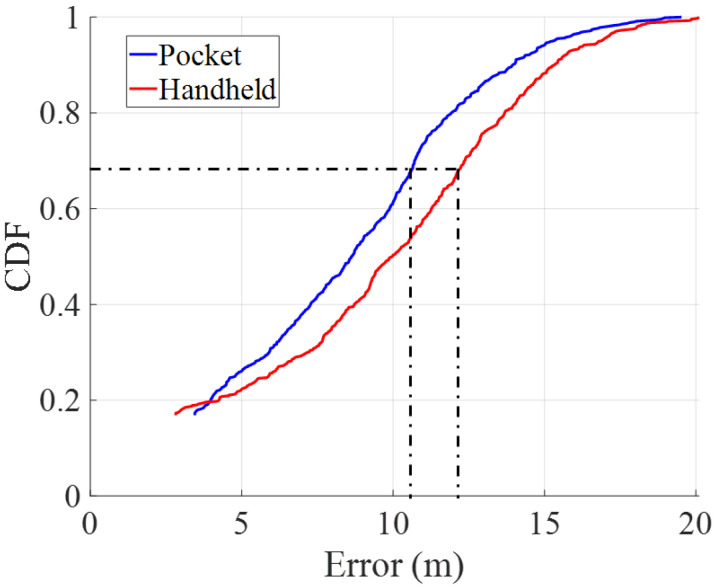
Cumulative density functions of the accumulated error for pocket (blue) and handheld (red) carrying modes with landmark-based position correction.

Figure 23 and Figure 24 show the cumulative distribution functions (CDFs) of the error for pocket and handheld smartphones without and with landmark-based correction, respectively. These CDFs have a minimum value that corresponds to the error at the first GTP of the walk.

We take as a reference the 1-σ value of the CDF to compare the position error between pocket and handheld carrying modes. The 1-σ value means that the error is bounded below a certain value in the 68% of the time.

In comparing the error between both carrying modes, the CDFs show that the pocket carrying mode is more accurate than the handheld carrying mode with and without landmark-based position correction. Table 2 summarizes the 1-σ error for both carrying modes.

The results show that there is approximately 23% reduction in the positioning error for the pocket carrying mode and 19% reduction for the handheld carrying mode. Therefore, the error reduction is more effective in the pocket carrying mode than in the handheld carrying mode. The results show that the smartphone in pocket carrying mode accumulates less error. Furthermore, the landmark-based position correction is more effective in the pocket carrying mode. This is due to the more effective detection of stairs in the pocket carrying mode, since the barometer-based detection of stairs misses some staircases that are 1.5 meters high or lower.

## 5. Conclusions

In this work we propose a smartphone-based localization system for passengers commuting in traffic hubs. We evaluate our system with more than 30 h of real measurements using mm-accuracy ground truth points measured in our test station in Munich city center. These ground truth points were visited over 3000 times to create the presented error curves. We demonstrate that it is possible to perform passenger localization in real-world stations with an average 3D accuracy of 11 m. These results are obtained without satellite aid, only using inertial and barometric sensors embedded in commercial smartphones.

We propose the use of stairs and lifts as landmarks to aid positioning in stations. We demonstrate that the proposed landmarks can be seamlessly detected with the smartphone while the passenger walks, and this reduces the positioning error in average 21%. 

## Figures and Tables

**Table 1:** Example of two landmarks stored in the database.

Name	Type	Upper Position	Lower Position	$\psi_{\mathrm{up}}$	$\psi_{\mathrm{down}}$
L1	Staircase	[10.4,−41.5,−3.6] m	[9.8,−47.2,−2.8] m	$95^{\circ }$	$275^{\circ }$
L2	Elevator	[−4.1,−30.1,−2.7] m	[−4.1,−30.1,−1.3] m	N/A	N/A

**Table 2:** Performance metrics of the smartphone localization systems for the pocket and handheld carrying modes before and after incorporating the landmark correction.

	No Landmark Correction	Landmark Correction
Pocket	σ=13.5 m	σ=10.5 m
Handheld	σ=14.7 m	σ=12 m
